# Maternal investment, life-history strategy of the offspring and adult chronic disease risk in South Asian women in the UK

**DOI:** 10.1093/emph/eow011

**Published:** 2016-04-09

**Authors:** Jonathan C.K. Wells, Pallas Yao, Jane E Williams, Rebecca Gayner

**Affiliations:** Childhood Nutrition Research Centre, UCL Institute of Child Health, 30 Guilford Street, London, WC 1N 1EH, UK

**Keywords:** life-history theory, blood pressure, growth, menarche, body composition, maternal investment

## Abstract

**Background and objectives**: Patterns of development predict cardiovascular disease (CVD) risk, and ethnic differences therein, but it remains unclear why apparently ‘adaptive plasticity’ in early life should generate health costs in later life. We hypothesized that offspring receiving low maternal investment during fetal life, the primary period of organogenesis, should predict a shorter reproductive career and develop a fast life-history strategy, prioritizing reproduction over growth and homeostatic maintenance.

**Methodology**: We studied 58 young adult South Asian women living in the UK, a group with high susceptibility to CVD. We obtained gestational age, birth weight (BW) and menarcheal age by recall and measured anthropometry, body composition, resting metabolic rate (RMR) and blood pressure (BP).

**Results**: BW and gestational age were inversely associated with menarcheal age, indicating that lower maternal investment is associated with faster maturation. Menarcheal age was positively associated with height but inversely with adiposity, indicating that rapid maturation prioritizes lipid stores over somatic growth. BW was inversely associated with BP, whereas adiposity was positively associated, indicating that lower maternal investment reduces BP homeostasis. BW was positively associated with RMR, whereas menarche was inversely associated, indicating that maternal investment influences adult metabolism.

**Conclusions and implications**: Supporting our hypothesis, low maternal investment promoted faster life histories, demonstrated by earlier menarche, reduced growth and elevated adiposity. These traits were associated with poorer BP regulation. This is the first study demonstrating strategic adjustment of the balance between reproduction and metabolic health in response to the level of maternal investment during fetal life.

## Abstract (precis)

We shows that women who received low maternal investment during fetal life, the primary period when the body's organs develop, develop a ‘fast’ life history strategy. This prioritizes reproduction (indicated by early menarche, higher body fatness) over somatic growth (adult height) and the ability to maintain healthy blood pressure.

## INTRODUCTION

Cardiovascular disease (CVD) is now the leading cause of death worldwide [1]. CVD is linked to a number of physiological risk factors that emerge progressively through the life-course, including hypertension, obesity, insulin resistance, dyslipidaemia and kidney dysfunction. Key risk factors in adult life include unhealthy diets, smoking, sedentary behaviour and obesity, particularly abdominal obesity [2].

There is now compelling evidence that patterns of growth and development mediate the associations of adult lifestyle and body composition with disease risk, which underpins the Developmental Origins of Health and Disease (DOHaD) hypothesis. Holding constant adult BMI and lifestyle factors, those with low birth weight (BW) have higher risk of hypertension, diabetes and ischaemic heart disease [3–5]. Rapid weight gain, especially after infancy, is another independent risk factor for increased fat deposition during childhood and elevated cardiometabolic risk [6]. Studies of non-human animals link rapid growth with markers of accelerated aging such as telomere attrition [7]. Variable growth patterns also contribute to ethnic variability in CVD risk [8].

The first conceptual model linking growth patterns with adult cardiovascular risk was the thrifty phenotype hypothesis of Hales and Barker [9]. This approach assumed that adaptation to fetal energy insufficiency, proxied by low BW, reduced the ability to tolerate ‘nutritional affluence’ in later life, hence promoting CVD risk. However, the inverse association between CVD risk and BW holds across the entire BW spectrum [3, 10], indicating that it cannot specifically represent the consequence of fetal ‘under-nutrition’. Even within the normal range of BW, greater BW reduces risk of hypertension and diabetes [5, 11], though macrosomic neonates may have elevated risks [12].

We have built on the ‘thrifty phenotype’ hypothesis to propose a ‘capacity-load’ model of disease risk, emphasizing two generic variables: (i) ‘metabolic capacity’, which scales directly with early-life growth and confers the capacity to maintain homeostasis in later life, and (ii) diverse stresses that collectively generate a ‘metabolic load’, exacerbating cardio-metabolic dysfunction [13]. Metabolic load incorporates various factors, that elevate oxidative stress and other modes of cellular deterioration, such as large body mass and obesity, lipogenic diets, sedentary behavior, smoking and inflammation. Support for this model is given by many epidemiological analyses showing that CVD risk is greatest in those born small (diminished capacity) who subsequently became large (high load) [6, 14, 15].

An evolutionary approach may shed more light on why, through the life-course, individuals acquire different capacity and load. Evolutionary ‘life-history theory’ assumes that energy must be allocated across four competing functions—‘maintenance’, ‘growth’, ‘reproduction’ and ‘immune function’ [16, 17]. Natural selection is assumed to have favoured energy allocation strategies that optimize lifetime reproductive fitness before health and longevity. For example, a study of rural Gambian women found that mean adult height (reflecting the duration of growth) was close to the value that would optimize the number of surviving offspring [18]. Through plasticity, each individual responds to ecological signals under this fitness-optimizing strategy, and through such responses, variability in each of metabolic capacity and load may emerge.

Individuals can be ranked along a continuum of ‘slow’ to ‘fast’ life-history strategies, according to the ecological cues encountered during development [19]. Slower life histories are favoured when the odds of survival are high, whereas faster life histories are favoured when mortality risk is high. Although uncontrollable or ‘extrinsic’ mortality risks are often assumed to shape the pace of life-history trajectory [20, 21], a little-explored hypothesis is that the intrinsic quality of the body may also be important [22, 23]. Body size at birth is a strong predictor of survival [24] and adult longevity [4, 25], hence early growth patterns are expected to shape life-history trajectory in similar ways to extrinsic mortality risk.

Given its central role in homeostasis, metabolic capacity can be considered a key component of long-term ‘maintenance’ [13]. Since it is strongly imprinted by nutritional experience during early ‘critical windows’, it indexes the magnitude of maternal investment during pregnancy and, to a lesser extent, lactation. We have argued previously that metabolic capacity develops specifically during critical windows that closely match the duration of maternal physiological investment [26]. On this basis, maternal investment, reflecting ‘maternal capital’, shapes the long-term quality of the offspring and hence the pace of its life history strategy [26–28].

Specifically, offspring receiving greater maternal investment during critical windows can convert it into greater metabolic capacity, predicting a longer lifespan and hence a longer reproductive career. Greater maternal investment favours a ‘slow life history’, with a lengthier period of growth, so that its physiological costs (e.g. telomere attrition) are minimized [7]. This further increases the likelihood of surviving long enough to reap the anticipated long-term reproductive pay-offs. The combination of high metabolic capacity and slow maturation is expected to reduce CVD risk in adult life, while the extended duration of growth increases final adult height.

Conversely, offspring receiving low maternal investment acquire limited metabolic capacity, whilst also experiencing oxidative stress [29]. These challenges to ‘maintenance’ predict a shorter lifespan and reproductive career. This scenario favours rapid growth and earlier maturation, leading to shorter adult height. Even though this may magnify the physiological costs of growth (e.g. telomere attrition), these can be partially discounted as the individual may not live long enough to pay them in full anyway [30]. After critical windows close, it is not possible to divert energy directly to metabolic capacity. Instead, any additional energy must be diverted to other components of maintenance, or to other life-history functions, such as growth, reproduction and immune function. Energy may be directly invested in these functions, or initially diverted to adipose tissue reserves, which can then release energy for these functions as required. Adiposity confer both fitness benefits (e.g. energy reserves for reproduction, immune function) but also costs (e.g. inflammatory load, hemodynamics stress etc) [31]. The combination of low metabolic capacity and high load (catch-up growth, adiposity, short adult stature) is expected to increase CVD risk in adult life.

**Figure 1.**
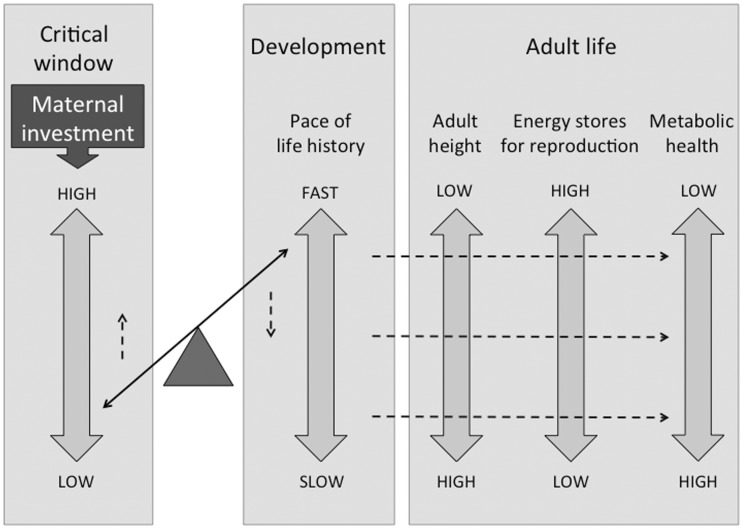
Schematic diagram illustrating how the level of maternal investment in fetal life is associated with the pace of maturation, which then mediates a trade-off between the capacities for reproduction versus maintaining metabolic homeostasis in adult life

These predictions are summarized in a schematic diagram in Fig. 1, showing how maternal investment in early life is expected to shape subsequent life-history ‘pace’, reproductive potential and cardio-metabolic risk of the offspring.

Among South Asians, rates of CVD are consistently higher relative to most other ethnic groups [32, 33]. Ethnic differences in growth patterns and body composition development are now thought to represent an important mechanism underlying the differential CVD risk. In both sexes, South Asians on average are shorter than Europeans, have less lean mass and more fat mass (FM), and greater central abdominal fat [33, 34]. Furthermore, this ‘thin-fat’ phenotype is already evident in early life [8, 35]. Our evolutionary perspective may help clarify ethnic variability in CVD risk.

Previous studies of Indian girls living in Sweden suggest that life-history traits may contribute to their elevated CVD risk. Born in India, they had low BW and were stunted, indicative of reduced metabolic capacity. In Sweden, they encountered a higher childhood energy supply, but were unable to translate this into larger adult size. Instead, they underwent very early puberty, which in due course led to short adult stature and elevated fatness [36]. The consequences of this accelerated pattern of development for CVD risk have not been described.

We therefore tested more comprehensively the hypothesis that the magnitude of maternal investment in early life shapes the life-history trajectory of the offspring, to provide a novel evolutionary perspective on the early-origins of adult CVD risk. We studied young adult South Asian women living in the UK, to gain greater insight into their elevated susceptibility to cardiovascular risk before the onset of reproduction and overt CVD. BW and gestational age were taken as markers of maternal nutritional investment. We then used age at menarche to assess the tempo of maturation, height and fat-free mass as growth outcomes, body fat and metabolic rate as the potential to invest in reproduction. We used blood pressure (BP) as an index of CVD risk, consistent with many other DOHaD studies [5, 14, 37].

## METHODS

### Subjects

We recruited healthy South Asian women in central London, UK, using posters, flyers in the local vicinity and email bulletin boards at local universities. Inclusion criteria were age between 18 and 30 years, gestational age 37+ weeks, and four South Asian grandparents. We excluded twins, smokers, pregnant/lactating women, weight instability (>3 kg change in the previous 3 months) and those with medical conditions known to impact body composition or metabolism. The study involved a 2.5-h measurement session, conducted at Great Ormond Street Hospital, London, UK. Participants were offered a cash payment of £10 following the measurements, as a token of appreciation for their time. Ethical approval was given by UCL Graduate School Ethics Committee. Written informed consent was obtained.

### Maternal residence and ethnicity

We obtained information on parental ethnicity, the number of generations of residence in the UK, and for first-generation migrants, duration of residence in the UK.

### Maternal investment and rate of maturation

BW, gestational age, infant feeding mode and the duration of breastfeeding were obtained through recall using a customised questionnaire. Participants were asked to contact their mothers to obtain this information. It was not possible to verify it from other records. The data were combined to give BW standard deviation score (SDS), adjusting for gender and gestational age, using UK 1990 reference data [38]. Age at menarche was also obtained by recall.

### Anthropometry and body composition

Height and sitting height were measured to the nearest 0.1 cm in duplicate, with the head in the Frankfort Plane. Leg length (LL) was calculated as the difference between sitting height and height. Waist girth was measured to the nearest 0.1 cm in duplicate with a non-stretchable tape.

Weight and body composition were assessed in duplicate using air-displacement plethysmography (Bodpod instrumentation; Cosmed USA), as described in detail previously [39]. Subjects wore a lightweight close-fitting swimming costume. Lung volumes were predicted using adult-specific equations. Raw values for body density are converted to body composition using Archimedes principle.

Body mass index (BMI, kg/m^2^) was calculated as weight/height^2^. To express whole-body adiposity adjusted for size but independent of fat-free mass (FFM), fat mass (kg) was divided by height^2^ to give the fat mass index (FMI, kg/m^2^) [40].

### Resting metabolic rate and BP

Resting metabolic rate (RMR) was measured in a thermoneutral environment after overnight fast using a Deltatrac Mk1 metabolic monitor. We have previously demonstrated high accuracy and precision of this instrumentation [41]. The monitor was calibrated using reference gases prior to every measurement. The subject lay supine and awake on a couch. A perspex hood collected expired respiratory gases, and analysed them for oxygen and carbon dioxide content on a minute by minute basis. Data were initially discarded until the subject had reached a stable state, and then collected for ≥10 min. Energy expenditure was calculated using Weir’s equation [42].

Systolic blood pressure (SBP) and diastolic blood pressure (DBP) were measured in duplicate with an automated oscillometric device (Accutorr, Datascope Corp, NJ), using an appropriate cuff size. Measurements were made after a few minutes rest, with the subject seated, feet flat on the floor.

### Conceptual approach

We hypothesized that daughters should strategically adjust their developmental trajectory in relation to the level of maternal investment during fetal life, proxied by BW SDS, generating trade-offs between maturation/adiposity versus growth/maintenance as described in the Introduction. This directly links the maternal capital hypothesis [28] with the capacity-load model of CVD risk [13]. Since the number of nephrons in the kidney is critical to long-term BP regulation, and since nephron number is fixed by birth [43], BW acts as a signal of how much the mother has invested in her offspring’s capacity for homeostatic maintenance. This allows us to focus specifically on maternal investment during fetal life. For many traits, however, plasticity continues into infancy, hence we also took into account breast-feeding duration as a simple index of post-natal maternal nutritional investment.

### Statistical analyses

Preliminary analyses tested for variability in maternal/participant characteristics according to generation of migration, using ANOVA and chi-squared test. Independent-sample *t*-tests and chi-squared tests were also used to test potential differences between those born in the UK, and those born overseas.

Regression analysis was used to investigate associations of BW and gestational age with age at menarche, to test the hypothesis that the duration and magnitude of maternal investment shaped the subsequent rate of maturation.

Regression analysis tested whether variability in (i) the magnitude of maternal investment, proxied by birth weight SDS, and (ii) maturation rate, proxied by menarcheal age, predicted adult size (stature, LL) and body composition. Due to non-normal distribution, adiposity outcomes were natural log-transformed.

Regression analysis further tested whether variability in (i) birth weight SDS, and (ii) maturation rate or adult tissue masses predicted adult BP. This is equivalent to testing the ‘capacity-load’ model, in which birth weight SDS represents metabolic capacity and adult tissue masses represent metabolic load. We also tested associations of birth weight SDS, adult body composition and menarcheal age with RMR, to elucidate developmental influences on adult metabolism.

Since growing up in the UK versus South Asia might affect the response to maternal investment, first-generation migration (coded as dummy variable) was treated as a potential confounding factor in all regression models. Breast-feeding duration (months) and first-born status (dummy variable), both of which may be considered as components of maternal reproductive strategy, were treated as potential mediating factors. The cut-off for statistical significance was *P* = 0.05, but due to the small sample size, confounders/mediators were included in models if *P* < 0.1.

## RESULTS

There were 210 respondents to recruitment. Of these, 62 women (30%) participated, the others failing to meet inclusion/exclusion criteria. Due to missing data on BW (*n* = 3) or gestational age (*n* = 2), birth weight SDS could be calculated for only 58 subjects. All analyses described below refer to this sample.

Of those analyzed, 14 were first-generation migrants, 33 second-generation and 11 third-generation. Of those born overseas, average residence in the UK was 3.2 years (range 0.25–13 years). Most participants were attending local academic institutions (*n* = 51), with the remainder in full time employment. There were 35 participants of Indian origin, 14 Sri Lankan, 7 Bangladeshi, 2 Pakistani, 1 Nepalese and 3 of mixed South Asian backgrounds.

The characteristics of the sample are shown in Table 1. The majority (95%) had been breast-fed (median 6 months, interquartile range 3–12 months). Only one subject was born preterm (36 weeks) according to conventional criteria (≤36 weeks), but she was not excluded as average of pregnancy duration is ∼1 week shorter in Asian compared to European populations [44]. There was a non-significant difference in birth weight SDS (Δ = 0.33 SD, 95%CI −0.30, 0.96) between firstborns (*n* = 22) and later-borns (*n* = 36). Firstborn status was not significant in any models described below. Using the obesity cut-off proposed for the Indian population (25 kg/m^2^), 13 women (21%) were obese, but BMI also varied widely (17.0–35.4 kg/m^2^).

**Table 1.** Description of the sample (*n* = 58)

	Mean	Standard deviation	Range
Development			
Birth weight (kg)	3.14	0.53	1.90, 4.59
Gestational age (weeks)	39.4	1.4	36, 42
Birth weight SDS	-0.34	1.14	-2.78, 2.27
Age at menarche (years)	12.4	1.7	9, 16
Adulthood			
Age (years)	22.6	3.4	18, 30
Weight (kg)	58.8	13.0	41.1, 113.6
Height (cm)	161.9	5.6	148.4, 179.1
Sitting height (cm)	84.7	3.0	76.3, 90.1
Leg length (cm)	77.3	3.7	71.1, 89.8
Waist girth (cm)	71.7	9.0	57.2, 95.4
BMI (kg/m^2^)	22.3	4.1	17.0, 35.4
Triceps skinfold (mm)	20.3	5.6	11.7, 34.1
Subscapular skinfold (mm)	18.7	7.2	7.6, 36.3
Fat-free mass (kg)	45.9	4.0	37.1, 57.1
Fat mass (kg)	12.9	10.0	3.9, 65.7
Systolic blood pressure (mmHg)	108.2	8.8	90.7, 127.2
Diastolic blood pressure (mmHg)	69.9	6.0	56.5, 81.5
Resting energy expenditure (kcal/day)	1350	160	1070, 1379

BMI, body mass index ; SDS, standard deviation score.

Variability in life-history and maternal traits stratified by migration status are given in Supplementary Table 1. Mothers of second-generation migrants were significantly older and of borderline-significant shorter stature, while third-generation migrants were significantly less likely to be first-borns. However, migration status was not associated with birth weight SDS, breast-feeding duration, age at menarche or adult height.

**Figure 2.**
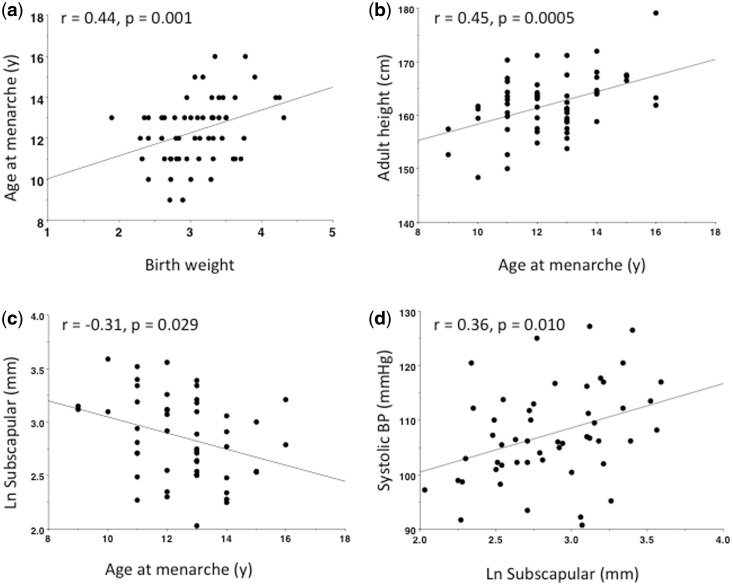
Empirical associations between maternal investment (proxied by birth size), maturation rate and adult phenotype. (**a**) Birth weight is positively associated with age at menarche. (**b**) Earlier menarche is associated with lower adult stature. (**c**) Earlier menarche is associated with higher adult subscapular skinfold. (**d**) Subscapular skinfold is positively associated with adult systolic blood pressure.

Table 2 describes regression models investigating associations of BW, gestational age and age at menarche with height, LL and body composition. Both gestational age (ß = 0.44 years, 95% CI 0.15, 0.74) and BW (ß = 0.49 years, 95% CI 0.14, 0.84) were positively associated with age at menarche (Fig. 2a), indicating that greater levels of maternal investment were associated with a slower life-history trajectory. In all subsequent models, gestational age was not independently significant, hence birth weight SDS was analyzed.

**Table 2.** Developmental predictors of maturation schedule and adult size

Outcome	Predictors	B	SE	*P*	*r*^2^
Menarche (y)	Constant	−4.914	5.764	0.3	0.186
	Birth weight SDS	0.491	0.177	0.007	
	Gestation (wks)	0.444	0.146	0.004	
Height (cm)	Constant	147.91	5.052	<0.0001	0.286
	Birth weight SDS	1.709	0.578	0.005	
	Menarche (y)	1.176	0.399	0.005	
Sitting height (cm)	Constant	82.460	3.063	<0.0001	0.099
	Birth weight SDS	0.833	0.351	0.021	
	Menarche (y)	0.201	0.242	0.4	
Leg length (cm)	Constant	65.451	3.383	<0.0001	0.293
	Birth weight SDS	0.876	0.387	0.028	
	Menarche (y)	0.975	0.267	0.001	
Fat free mass (kg)	Constant	47.636	3.993	<0.0001	0.063
	Birth weight SDS	1.087	0.457	0.021	
	Menarche (y)	−0.111	0.315	0.7	
Ln triceps (mm)	Constant	3.441	0.280	<0.0001	0.112
	Birth weight SDS	0.064	0.033	0.058	
	Menarche (y)	−0.039	0.022	0.083	
	First-generation	0.210	0.084	0.015	
Ln subscapular (mm)	Constant	3.679	0.424	<0.0001	0.175
	Birth weight SDS	−0.021	0.047	0.6	
	Menarche (y)	−0. 076	0.033	0.026	
	Breast-feeding (m)	0.017	0.007	0.015	
Ln waist girth (cm)	Constant	4.464	0.126	<0.0001	0.059
	Birth weight SDS	0.011	0.015	0.4	
	Menarche (y)	−0.018	0.010	0.073	
	Breast-feeding (m)	0.004	0.002	0.090	

Birth weight SDS (ß = 1.7 cm, 95% CI 0.55, 2.87) and age at menarche (ß = 1.2 cm, 95% CI 0.4, 2.0) were each positively associated with height (Fig. 2b), and also with relative LL, but only birth weight SDS was associated with sitting height. Thus, a slower life-history trajectory, indicated by greater fetal growth and later sexual maturation, was associated with greater completed growth, and during post-natal life this association was restricted to growth of the lower limb. Birth weight SDS was significantly associated with FFM (ß = 1.09 kg, 95% CI 0.17, 2.00), whereas menarcheal age was not predictive. Indices of adiposity were negatively associated with menarcheal age (Fig. 2c), though this association only achieved significance for the subscapular skinfold (ß = −1.5 mm, 95% CI −2.8, −0.2), whereas birth weight SDS was not predictive. These models took into account first-generation migrants having greater Ln triceps (ß = 0.19 mm, 95% CI 0.03, 0.36) than other groups, and breast-feeding duration (months) being positively associated with Ln subscapular skinfold (ß = 0.02 mm, 95% CI 0.00, 0.03) and Ln waist girth. Thus a faster life-history trajectory was associated with relatively greater adiposity, but an accelerated pace of maturation after birth appeared more important in this association than reduced weight gain during fetal life.

Table 3 describes associations between birth weight SDS and body composition with BP, testing the ‘capacity-load’ model. Birth weight SDS consistently showed inverse associations with BP, although the relationship was only significant for DBP (ß = −1.3 mmHg, 95% CI −0.0, −2.7), when the index of adiposity was Ln FMI (ß = 4.3 mmHg, 95% CI 1.7, 6.9). Indices of body fat were positively associated with both SBP (Fig. 2d) and DBP, significant in almost all models. In general, the indices of central fat (Ln waist girth (DBP: ß = 20.9 mmHg, 95% CI 8.9, 32.9); Ln subscapular skinfold (DBP: ß = 6.1 mmHg, 95% CI 2.0, 10.2) showed stronger associations with BP than our index of peripheral fat (Ln triceps skinfold: DBP: ß = 2.9 mmHg, 95% CI −0.1, 11.4). Age at menarche was not significant in these models.

**Table 3.** Capacity-load models of blood pressure and RMR

Outcome	Predictors	B	SE	p	r^2^
Systolic BP (mmHg)	Constant	99.345	2.990	<0.0001	0.130
	Birth weight SDS	−1.599	0.981	0.11	
	Ln FMI (kg/m^2^)	5.959	1.939	0.003	
	Constant	93.101	13.074	<0.0001	0.007
	Birth weight SDS	−1.331	1.067	0.2	
	Ln Triceps (mm)	4.885	4.354	0.2	
	Constant	84.943	8.666	<0.0001	0.124
	Birth weight SDS	−1.367	1.031	0.19	
	Ln Subscapular (mm)	7.672	3.021	0.014	
	Constant	−7.334	38.803	0.8	0.122
	Birth weight SDS	−1.318	0.973	0.18	
	Ln Waist (cm)	26.986	9.087	0.004	
Diastolic BP (mmHg)	Constant	63.432	1.999	<0.0001	0.165
	Birth weight SDS	−1.345	0.656	0.045	
	Ln FMI (kg/m^2^)	4.347	1.296	0.001	
	Constant	52.694	8.651	<0.0001	0.066
	Birth weight SDS	−1.240	0.706	0.085	
	Ln Triceps (mm)	5.629	2.881	0.056	
	Constant	51.376	5.857	<0.0001	0.184
	Birth weight SDS	−1.183	0.697	0.096	
	Ln Subscapular (mm)	6.133	2.042	0.004	
	Constant	19.641	25.645	0.4	0.176
	Birth weight SDS	−1.154	0.643	0.078	
	Ln Waist (cm)	20.916	6.006	0.001	
RMR (kcal/day)	Constant	−2300.1	571.4	<0.0001	0.444
	Birth weight SDS	23.55	14.30	0.106	
	Ln Waist (cm)	861.56	134.00	<0.0001	
	First-generation	−66.63	37.65	0.082	
	Constant	−1768.3	634.1	0.007	0.462
	Fat free mass (kg)	12.712	5.933	0.037	
	Ln Waist (cm)	598.42	187.67	0.002	
	First-generation	−70.39	36.95	0.062	
	Constant	−3085.2	600.6	<0.0001	0.489
	Menarche (y)	24.77	9.74	0.014	
	Ln Waist (cm)	971.66	132.19	<0.0001	
	First-generation	−77.19	36.53	0.039	

BP, blood pressure; FMI, fat mass index; RMR, resting metabolic rate; SDS, standard deviation score

In DBP regression models, breast-feeding duration was a mediating factor (Supplementary Table 2). Its inclusion reduced the ß-coefficients of adiposity variables (e.g. Ln subscapular: ß declined from 6.1 (95% CI 2.0, 10.2) to 4.8 (95% CI 0.5, 9.1). In contrast, birth weight SDS coefficients were unchanged, but their *P* values increased slightly due to the reduction in degrees of freedom.

Birth weight SDS was positively associated with RMR with borderline significance (ß = 35 kcal/day, 95% CI −2, 72), however it was displaced from models that included either adult height or FFM, indicating that the BW association was explained by its association with adult size (birth weight SDS and FFM, *r* = 0.31, *P* = 0.018). Adjusting for FFM, Ln waist girth was positively associated with RMR (ß = 573 kcal/day, 95% CI 189, 957). Adjusting for waist girth, age at menarche was positively associated with RMR (ß = 22 kcal/day, 95% CI 3, 43), attributable to the positive association of age at menarche with stature and FFM. In these models, first-generation migration was also significant (e.g. FFM model, ß = −79 kcal/day, 95% CI −7, −150), indicating lower BMR in those born in South Asia rather than the UK.

## DISCUSSION

We tested the hypothesis that maternal investment in fetal life would shape the subsequent life-history trajectory of female offspring, and that lower maternal investment would accelerate the offspring’s maturation and skew her subsequent investment towards reproduction at the cost of growth and maintenance. Our findings support each component of this hypothesis: less maternal investment in fetal life was associated with faster maturation, reduced completed growth, elevated adiposity and poorer BP regulation. Of particular interest, both indices of maternal investment (BW for gestational age, and the duration of gestation itself) predicted the post-natal rate of maturation of the offspring.

In our approach, we treat both lower BW and earlier menarche as markers of a faster life history. However, the second of these factors appears especially important for long-term phenotype. In the absence of catch-up growth, studies repeatedly indicate that the metabolic consequences of low BW are modest. For example, in a study of ∼80 000 women, menarche occurred only ∼2 months earlier in those with the lowest relative to the highest BWs, but a year earlier in those of high versus low weight at 7 years, regardless of BW [45]. Likewise, low BW only predicts high BP in those with high metabolic load in later life [5, 14].

We found that a slower life-history trajectory is associated with higher RMR, due to larger FFM. However, this may offer fitness benefits, since large body size increases the energetic efficiency of reproduction [46], while BW of the offspring scales directly with maternal RMR during pregnancy [47]. Larger mothers (i.e. those with greater somatic capital) are able to pass these traits on to the next generation [48]. Conversely, mothers with fast life histories also pass the relevant traits to the next generation. In the ALSPAC cohort, for example short fat mothers with early menarche had daughters who developed the same traits, mediated by rapid infant growth [49]. Intriguingly, both maternal adiposity and RMR promote maternal investment in the offspring, but RMR may be more favourable for nutritional investment *in utero*, when the ‘maintenance’ benefits for the offspring are greatest. An intriguing finding was the lower RMR of those born overseas, which we speculate might reflect the imprinting of metabolism by thermal conditions during early life.

We focused on an index of CVD risk that is particularly sensitive to fetal growth. For BP, a key index of metabolic capacity is nephron number, which cannot change after birth. Throughout post-natal life, weight gain is associated with higher BP [50]. We found that breast-feeding duration mediated this affect, so that through its association with adult adiposity, it also correlated with DBP. This is consistent with previous findings [51], and is likely to arise through the obesogenic niche amplifying an adaptive tendency for breast-feeding to promote the early acquisition of energy stores for female reproduction.

These findings provide an integrated evolutionary life-history perspective on the link between nutritional experience in early life and susceptibility to chronic disease risk in later life. Early plasticity is widely assumed to represent a component of adaptation, but why such plasticity, if initially adaptive, should provoke health costs in later life has remained controversial.

One proposed evolutionary explanation for the long-term metabolic consequences of variability in BW was the ‘predictive adaptive response’ hypothesis of Gluckman and colleagues [52, 53]. According to this approach, fetuses exposed to poor nutritional environments respond adaptively by anticipating similar harsh conditions in adult life, and prepare their physiology accordingly. Overt disease would occur if this prediction failed, resulting in the individual being metabolically ‘mismatched’ to their actual environment. This ‘adaptation through matching’ hypothesis has been extensively criticized for a number of reasons. First, there is no evidence that the long-term physiological consequences of low BW, most notably small body size, are indeed adaptive for survival or reproduction in adult life in any conditions. Rather, those born small are least likely to survive and reproduce during famines in adult life, suggesting no benefits of anticipatory ‘matching’ [54]. Second, in the absence of the obesogenic niche, those born small do not develop traits such as insulin resistance or hypertension, which were proposed to be adaptive in adult famine conditions [55].

We proposed a competing ‘maternal capital’ model, emphasizing that the human fetus is not directly exposed to the environment, so that any metabolic adaptation in early life is shaped by maternal physiology. We argued that the offspring initially adapts to the level of maternal investment in early life, and develops an appropriate life-history strategy accordingly [26, 28, 55]. The lower the level of maternal investment, the lower the homeostatic capacity of the body, and hence the shorter the expected lifespan. Since plasticity declines substantially at the end of infancy (which we have attributed to the withdrawal of maternal nutritional buffering [26]), the offspring must adapt by selecting a ‘pace’ of development that modifies the onset of reproductive maturity in association with its intrinsic somatic quality, indicative of longevity and the duration of the reproductive career. We predicted that low maternal investment would induce a faster pace of life history, diverting energy to reproduction at the expense of investment in maintenance [22]. This would translate into higher susceptibility to chronic disease, however this susceptibility would remain relatively latent until exposure to the obesogenic niche. This is addressed by our ‘capacity-load model’ of chronic disease risk, which predicts that low metabolic capacity only predisposes to chronic diseases if metabolic load is elevated [13].

Consistent with this hypothesis and with other studies [5, 14, 37], high levels of adiposity and low BW—each components of a fast life-history strategy—were each associated with higher DBP. Thus, in this population, low maternal investment induces not only a low metabolic capacity, through reduced fetal growth, but also elevated metabolic load through early menarche, associated with elevated adiposity, and hence poorer homeostatic function [49]. We also found, consistent with other studies [56], that central fat was more strongly associated than peripheral fat with DBP. Central fat is more closely linked with immune function [57], hence it represents a life-history strategy with short-term benefits in pathogen-rich environments, but also with long-term costs if disease exposure is low and energy supply favourable, allowing the fat depot to expand [58].

Our findings are consistent with previous studies of Indian girls who migrated to Sweden during early childhood. Born in India, these girls were characterized by low BW and by stunting on arrival in Sweden. Subsequent catch up growth did not fully resolve this stunting, and was also associated with early menarche compared to Swedish girls [36]. This scenario may however be restricted to populations that are able to accelerate their life-history trajectory during childhood, in compensation for poor fetal growth. Although our sample included women born both in the UK and overseas, the cohort represent an educated middle-class population, who have likely all experienced relatively favourable conditions since birth. Among chronically under-nourished populations, however, there may be no opportunity for catch up growth and accelerated maturation. For example, in many low-income countries, low social status groups experience later menarche than high social status groups [59].

Many authors have emphasized that *extrinsic* mortality risk should shape life-history trajectory [18, 20, 21]. This is supported by comparisons both across and within species [19]. However, variability in life-history trajectory is still apparent in populations where mortality risk is generically low, suggesting that other factors are also important. Ours is the first study to demonstrate a link between poorer *intrinsic* quality of the body (reflecting low maternal investment during the key period of organogenesis), accelerated reproductive maturation, and poorer homeostatic capacity in later life. This is consistent with life-history perspectives on obesity [22] and mathematical models of developmental strategy [23].

Our approach helps explain why ethnic groups chronically exposed to low-energy high-pathogen environments have elevated CVD risk following migration to high-energy low-pathogen environments. Notably, South Asian BWs have increased negligibly across generations in the UK [60], indicating persistent constraint on metabolic capacity. Any surplus energy may therefore be shunted to rapid maturation and reproduction, rather than growth/maintenance as described above. On exposure to obesogenic environments following rural-urban or overseas migration, South Asian populations are therefore susceptible to a double whammy of elevated metabolic load and depleted metabolic capacity, a scenario known as the ‘thin-fat’ phenotype [8, 34].

Our study had several limitations, including relatively small sample size, use of recall methods to obtain data on gestational age, BW and age at menarche, and lack of direct data on maternal capital (e.g. body size, energy stores). We were however able to adjust for the number of generations residence in the UK, and post-natal nutritional investment proxied by breast-feeding duration. The strengths of the study included the wide range of outcomes, allowing us to connect life-history strategy with each of growth, reproductive maturation, body composition measured using a reference method, and chronic disease risk.

In summary, this study provides a life-history perspective on the association of nutrition in early life with later chronic disease risk. Linking the ‘maternal capital’ and ‘capacity-load’ models [13, 28], we propose that low maternal investment, the principle ecological ‘stress’ directly experienced during fetal life, steers the offspring towards a faster life-history trajectory, though the magnitude of this adaptive response depends strongly on the quality of the childhood environment. In consequence, in a high-energy environment, the offspring matures rapidly with elevated fat stores for reproduction, but at a cost of reduced growth and depleted homeostatic capacity for BP regulation. This strategy is ultimately adaptive, since reproductive fitness is enhanced in the short-term, while the potential costs of overt chronic disease risk lie later in life, and may never be paid. However, fitness may be maximized at the cost of health. Our approach may be especially valuable for understanding ethnic variability in CVD risk.

## FUNDING 

This research was supported by the National Institute for Health Research Biomedical Research Centre at Great Ormond Street Hospital for Children NHS Foundation Trust and University College London.

**Conflict of interest**: None declared.

## Supplementary data

Supplementary data is available at EMPH online.

Supplementary Data
