# Supplementary material for: Maternal investment, life-history strategy of the offspring and adult chronic disease risk in South Asian women in the UK
Source: Evol Med Public Health. 2016 Apr 9;2016(1):133–45. doi: 10.1093/emph/eow011 (PMC4826584; doi:10.1093/emph/eow011)
Supplement: Supplementary Data [file supp_2016_1_133__index.html]

Supplementary Data 

# Maternal investment, life-history strategy of the offspring and adult chronic disease risk in South Asian women in the UK

## Supplementary Data

files

- Supplementary Data - docx file
