# Supplementary material for: Maternal investment, life-history strategy of the offspring and adult chronic disease risk in South Asian women in the UK
Source: Evol Med Public Health. 2016 Apr 9;2016(1):133–45. doi: 10.1093/emph/eow011 (PMC4826584; doi:10.1093/emph/eow011)
Supplement: Supplementary Data [file supp_eow011_SupplementaryTables.docx]

**Supplementary online Table 1. Sample characteristics stratified by generation of migration**

| **Generations in UK** | First-generation | | Second-generation | | Third-generation | | ANOVA  p-value | T-test  p-value |
| --- | --- | --- | --- | --- | --- | --- | --- | --- |
|  | N=14 | | N=33 | | N=11 | |  |  |
| **Outcome** | Mean | SD | Mean | SD | Mean | SD |  |  |
| Maternal age (year) * | 28.6 | 5.6 | 31.1 | 3.5 | 27.8 | 3.8 | 0.047 | 0.2 |
| Maternal height (cm) * | 161.6 | 6.9 | 157.4 | 4.8 | 161.6 | 5.6 | 0.055 | 0.2 |
| Birth weight SDS * | -0.46 | 1.3 | -0.15 | 1.12 | -0.76 | 0.87 | 0.29 | 0.6 |
| Age at menarche (years) * | 12.6 | 0.9 | 12.4 | 1.9 | 12.2 | 1.8 | 0.79 | 0.4 |
| Adult height (cm) * | 162.8 | 4.6 | 162.1 | 6.0 | 160.4 | 5.4 | 0.56 | 0.5 |
|  |  |  |  |  |  |  |  |  |
|  | Median | IQR | Median | IQR | Median | IQR |  |  |
| Breastfeeding (months)* | 4.5 | 1.5, 15.0 | 6.0 | 4.0,12.0 | 3.0 | 1.7, 6.1 | 0.18 | 0.8 |
|  |  |  |  |  |  |  |  |  |
|  | yes | no | yes | no | yes | no |  |  |
| Firstborn (frequency) ** | 13 | 1 | 22 | 11 | 3 | 8 | 0.003 | 0.013 |
|  |  |  |  |  |  |  |  |  |

* tested across 3 groups by one-way ANOVA, and across 2 groups (first vs second/third generation) by independent samples T-test

** tested across 2 and 3 groups by chi-square test

SDS – standard deviation score

**Supplementary Online Table 2. Capacity-load models of blood pressure and resting metabolic rate, adjusting for breast-feeding duration**

| Outcome | Predictors | B | SE | p | r^2^ |
| --- | --- | --- | --- | --- | --- |
| Diastolic BP (mmHg) | Constant | 62.791 | 2.036 | <0.0001 | 0.211 |
|  | Birth weight SDS | -1.310 | 0.668 | 0.055 |  |
|  | Ln FMI (kg/m^2^) | 3.663 | 1.362 | 0.010 |  |
|  | Breast-feeding (months) | 0.208 | 0.101 | 0.044 |  |
|  |  |  |  |  |  |
|  | Constant | 53.993 | 8.368 | <0.0001 | 0.144 |
|  | Birth weight SDS | -1.230 | 0.698 | 0.084 |  |
|  | Ln Triceps (mm) | 4.567 | 2.817 | 0.111 |  |
|  | Breast-feeding (months) | 0.245 | 0.104 | 0.022 |  |
|  |  |  |  |  |  |
|  | Constant | 53.663 | 5.947 | <0.0001 | 0.184 |
|  | Birth weight SDS | -1.182 | 0.705 | 0.101 |  |
|  | Ln Subscapular (mm) | 4.783 | 2.144 | 0.031 |  |
|  | Breast-feeding (months) | 0.200 | 0.105 | 0.064 |  |
|  |  |  |  |  |  |
|  | Constant | -8.716 | 26.507 | 0.7 | 0.227 |
|  | Birth weight SDS | -1.127 | 0.656 | 0.092 |  |
|  | Ln Waist (cm) | 17.963 | 6.248 | 0.006 |  |
|  | Breast-feeding (months) | 0.214 | 0.099 | 0.036 |  |
|  |  |  |  |  |  |

BP – blood pressure; SDS – standard deviation score; FMI – fat mass index
